# Different Influences of Hematocrit on the Results of Two Point-Of-Care Platelet Function Tests, the VerifyNow Assay and Multiple Electrode Platelet Aggregometry

**DOI:** 10.1371/journal.pone.0114053

**Published:** 2014-11-26

**Authors:** Yun Gi Kim, Jung-Won Suh, Jin Joo Park, Il-Young Oh, Chang-Hwan Yoon, Young-Seok Cho, Tae-Jin Youn, In-Ho Chae, Dong-Ju Choi

**Affiliations:** Cardiovascular Center, Seoul National University Bundang Hospital, Seongnam, Gyeonggi-do, Korea; West German Cancer Center, Germany

## Abstract

**Objective:**

Previous studies have reported a considerable association between the VerifyNow (Accumetrics, San Diego, CA, USA) P2Y12 assay results and hematocrit. No reports, however, have described an association between the multiple electrode platelet aggregometry (MEA; Dynabyte, Munich, Germany) adenosine diphosphate (ADP) assay results and hematocrit. This study was conducted to evaluate the influence of hematocrit on the results of 2 different point-of-care platelet function tests.

**Methods:**

A total of 462 consecutive patients who were undergoing percutaneous coronary intervention were enrolled. Platelet function was evaluated with both the VerifyNow P2Y12 and MEA ADP assays.

**Results:**

Anemic patients (n = 152, 32.9%) demonstrated a significantly higher rate of cardiac death, myocardial infarction, and stroke (5.3% vs. 2.3%, *p* = 0.046) during the follow-up (median: 18.8 months). Although the VerifyNow P2Y12 assay results demonstrated a significant inverse correlation with hematocrit (r = −0.409, *p*<0.001), there was no such correlation between the MEA ADP assay results and hematocrit (r = 0.039, *p* = 0.401). In the multivariate analysis, anemia was an independent predictor of high on-treatment platelet reactivity, defined as a VerifyNow P2Y12 reaction unit level of ≥252.5 (odds ratio = 2.21, 95% confidence interval = 1.39–3.52; *p* = 0.001). Importantly, this association was independent of an intrinsic change in platelet reactivity as measured by the MEA ADP assay. Adjusting for the influence of hematocrit improved the strength of the correlation between the VerifyNow P2Y12 and MEA ADP assay results.

**Conclusions:**

Hematocrit significantly influenced the VerifyNow P2Y12 assay results, a phenomenon that was presumably in-vitro. Hematocrit level should therefore be considered when interpreting results of the VerifyNow P2Y12 assay.

## Introduction

The VerifyNow (Accumetrics, San Diego, CA, USA) P2Y12 and multiple electrode platelet aggregometry (MEA; Dynabyte, Munich, Germany) adenosine diphosphate (ADP) assays are both point-of-care platelet function tests that evaluates the efficacy of ADP-receptor antagonists such as clopidogrel. A turbidimetric-based optical detection system is used for the VerifyNow assay and the principles of impedance aggregometry are applied in the MEA assay. [1] Several previous studies have reported a considerable association between the VerifyNow P2Y12 assay results and hematocrit level. [2]–[4] Toma et al. demonstrated that anemic patients had higher VerifyNow P2Y12 reaction unit (PRU) levels. [5] As anemia has been associated with adverse clinical outcomes in acute coronary syndrome patients, [6],[7] it is important to distinguish whether the observed association between the VerifyNow P2Y12 assay results and hematocrit is truly an in-vivo effect that represents an actual hematocrit-dependent intrinsic change in platelet reactivity or merely a laboratory artifact. A recent study reported that the effect of hematocrit on the VerifyNow P2Y12 assay results was just an in-vitro phenomenon that was independent of an intrinsic change in platelet reactivity. [2] However, there have been no reports to date whether the MEA ADP assay results are correlated with hematocrit. The aim of this study was to evaluate the influence of hematocrit on the results of 2 different point-of-care platelet function tests, the VerifyNow P2Y12 and MEA ADP assays, and to elucidate its clinical implication.

## Methods

### Patients

This study was a retrospective study and a precise description of the study cohort has been provided elsewhere. [8] Briefly, a total of 462 consecutive patients with coronary artery disease (CAD) and planned percutaneous coronary intervention (PCI) were recruited from Seoul National University Bundang Hospital between February 2010 and December 2011. Sample size calculation to establish a powered analysis was not performed since this study was not a randomized clinical trial. The protocols of this study were consistent with the ethical guidelines of the 1975 Helsinki Declaration. The institutional review board of Seoul National University Bundang Hospital ensured appropriate ethical & bioethical conduct and specifically approved this study (approval number: B-1404-246-105). Written informed consent was not obtained because this was a retrospective study. Institutional review boards, however, has approved the waiver of written informed consent and patient records & information was anonymized and de-identified prior to the analysis.

### Interventional procedures

Patients took daily 100 mg of aspirin and 75 mg of clopidogrel for at least 7 days before undergoing PCI. Loading doses of aspirin (300 mg) and clopidogrel (300 mg) were administered to patients who had not taken aspirin or clopidogrel. Most patients (98.3%, 454/462) underwent drug-eluting stent implantation. After PCI, aspirin and clopidogrel were administered for at least 6 months. Coronary angiography and PCI were performed in accordance with the current standard guidelines. Intravenous heparin was administered during PCI.

### Blood sampling

For all patients, blood samples were collected before the initial PCI procedure. A complete blood cell count analysis was performed using Sysmex XE-2100 (Sysmex, Mundelein, IL, USA). In our study, anemia was defined as (i) hematocrit <39% for men or (ii) hematocrit <36% for women, according to the World Health Organization criteria. [7],[9],[10]

### Clinical outcomes

We compared the clinical outcomes according to the presence of anemia. For the analysis of clinical outcomes, the term “major adverse cardiac events” (MACE) was defined as a composite of cardiac death, myocardial infarction (MI), and stroke. The median follow-up duration was 18.8 months, and the follow-up loss rate was 1.3%. The cause of death was regarded as cardiovascular unless there was documented evidence of a clear non-cardiovascular cause. MI was defined according to published guidelines using universal definition. [11] Stroke was defined as a new focal neurologic deficit of vascular origin lasting for at least 24 hours that was proven to be non-hemorrhagic by either computed tomography or magnetic resonance imaging scanning.

### Platelet function tests

The residual platelet reactivity after ADP-receptor antagonist treatment was measured with both the VerifyNow P2Y12 and MEA ADP assays. Blood samples were obtained from patients 1 day after the initial PCI procedure and were collected in Greiner Bio-One 3.2% citrate Vacuette tubes (Greiner Bio-One, Kremsmünster, Austria) for VerifyNow and in 4.5-mL plastic tubes containing the anticoagulant lepirudin (25 µg/mL, Refludan, hirudin blood collection tubes; Dynabyte) for MEA. The blood samples were kept at room temperature for at least 30 minutes before platelet function testing. Both tests were performed according to the manufacturers' instructions. The results of the VerifyNow P2Y12 and MEA ADP assays were quantified in PRU and units (10 aggregation units*min = 1 unit) respectively.

### Statistical analysis

Statistical analysis was conducted using SPSS version 18.0 (SPSS Inc., Chicago, IL, USA). Values are expressed as means ± standard deviations or medians with interquartile ranges as appropriate. For the statistical analysis, categorical variables were compared using the chi-square test or Fisher's exact test as appropriate. The Kolmogorov–Smirnov test was used to test the normal distribution of continuous data. Normally distributed variables were compared using the unpaired t-test and non-normally distributed variables were compared using the Mann–Whitney U test. Survival data were estimated according to the Kaplan–Meier method, and the differences between groups were compared with the log-rank test. Spearman's rank correlation coefficient (r) was used to perform a correlation analysis. Prior to the multivariate analysis, a univariate linear regression analysis was performed to compare the VerifyNow P2Y12 assay and the laboratory/clinical variables. Variables with *p*<0.1 in the univariate analysis were included in the multivariate model. The multivariate logistic regression analysis model was built to evaluate the effect of anemia on antiplatelet responsiveness. All significance tests were 2-tailed, and *p* values <0.05 were considered to indicate statistical significance.

## Results

### Patient characteristics

The baseline clinical characteristics of the study patients are summarized in Table 1. During the study period, a total of 462 CAD patients treated with PCI were enrolled. Of these, 70.3% were men and 19.7% of all patients presented with acute coronary syndrome. The mean hematocrit values were 40.91%±4.27% in men and 36.35%±3.63% in women. The results of the VerifyNow P2Y12 and MEA ADP assays were non-normally distributed (1 sample Kolmogorov–Smirnov test: *p* = 0.025, *p*<0.001 respectively). The median values were 232.00 (160.00–293.00) PRU for VerifyNow P2Y12 and 21.00 (15.00–31.00) units for MEA ADP. A PRU value of ≥252.5 has been identified as an optimal cutoff value for predicting post-discharge atherothrombotic events in Koreans. [12] According to this cutoff value, 187 (40.5%) patients had high on-treatment platelet reactivity (HTPR) after ADP-receptor antagonist treatment.

**Table 1:** Baseline clinical characteristics of the study patients.

	Total (N = 462)
Age	67.00 (58.00–72.00)
Male sex	325 (70.3%)
BMI (kg/m^2^)	25.29 (23.41–27.42)
Diabetes mellitus	142 (30.7%)
On insulin therapy	28 (6.1%)
Hypertension	280 (60.6%)
Smoking history	217 (47.0%)
Previous PCI	83 (18.0%)
Previous CVA	6 (1.3%)
CAD presentation	
SA	371 (80.3%)
UA	55 (11.9%)
NSTEMI	19 (4.1%)
STEMI	17 (3.7%)
Baseline laboratory findings	
Hematocrit (%)	39.56±4.59
Platelet count (10^2^/mm^3^)	205.00 (174.00–240.00)
Creatinine (mg/dL)	0.83 (0.72–0.99)
HbA1c (%)	6.10 (5.70–6.90)
TC (mg/dL)	158.00 (135.75–183.00)
TG (mg/dL)	122.00 (88.75–170.50)
HDL (mg/dL)	41.00 (35.75–48.00)
LDL (mg/dL)	89.00 (69.00–112.00)
Platelet function tests	
VerifyNow P2Y12 (PRU)	232.00 (160.00–293.00)
MEA ADP (units)	21.00 (15.00–31.00)

BMI: body mass index; PCI: percutaneous coronary intervention; CVA: cerebrovascular accident; CAD: coronary artery disease; SA: stable angina; UA: unstable angina; NSTEMI: non-ST elevation myocardial infarction; STEMI: ST elevation myocardial infarction; TC: total cholesterol; TG: triglyceride; HDL: high-density lipoprotein; LDL: low-density lipoprotein; PRU: P2Y12 reaction units; MEA ADP: multiple electrode platelet aggregometry adenosine diphosphate test.

Based on the above-described criteria, 152 (32.9%) patients were classified as anemic. The baseline clinical characteristics of non-anemic and anemic patients are presented in Table 2. Anemic patients were significantly older and had a lower body mass index than non-anemic patients. Women were more likely to be anemic. Anemic patients had a higher prevalence of diabetes mellitus and hypertension but a lower prevalence of smoking history.

**Table 2:** Baseline clinical characteristics of the study patients with and without anemia.

	Patients without anemia (n = 310)	Patients with anemia (n = 152)	*p* value
Age	63.00 (55.00–70.00)	69.50 (63.00–74.00)	<0.001
Male sex	232 (74.8%)	93 (61.2%)	0.003
BMI (kg/m^2^)	25.56 (23.85–27.67)	24.47 (22.44–26.38)	<0.001
Diabetes mellitus	79 (25.5%)	63 (41.4%)	<0.001
Hypertension	174 (56.1%)	106 (69.7%)	0.005
Smoking history	159 (51.3)	58 (38.2)	0.008
Baseline laboratory findings			
Platelet count (10^2^/mm^3^)	206.00 (174.00–242.25)	199.00 (173.00–237.25)	0.479
Creatinine (mg/dL)	0.82 (0.72–0.95)	0.88 (0.73–1.15)	0.006
HbA1c (%)	6.10 (5.70–6.80)	6.20 (5.70–7.00)	0.405
TC (mg/dL)	164.00 (142.00–190.00)	149.00 (125.00–175.50)	<0.001
TG (mg/dL)	125.50 (93.00–182.75)	110.00 (78.00–141.00)	<0.001
HDL (mg/dL)	41.00 (35.25–48.00)	41.00 (36.00–48.00)	0.800
LDL (mg/dL)	94.00 (74.00–116.00)	79.00 (62.50–102.00)	<0.001
CAD presentation			
SA	252 (81.3%)	119 (78.3%)	0.446
UA	33 (10.6%)	22 (14.5%)	0.233
NSTEMI	13 (4.2%)	6 (3.9%)	0.900
STEMI	12 (3.9%)	5 (3.3%)	0.755
Lesion location			
LM	28 (9.0%)	11 (7.2%)	0.514
LAD	213 (68.7%)	95 (62.5%)	0.183
RI	6 (1.9%)	4 (2.6%)	0.736
LCx	83 (26.8%)	41 (27.0%)	0.964
RCA	102 (32.9%)	49 (32.2%)	0.886
Multivessel disease	188 (60.6)	102 (67.1%)	0.177
Comedication			
Aspirin	310 (100.0%)	152 (100.0%)	
ADP-receptor antagonist	310 (100.0%)	152 (100.0%)	
Cilostazol	69 (22.3%)	28 (18.4%)	0.341
Beta blocker	170 (54.8%)	83 (54.6%)	0.962
ACEI or ARB	211 (68.1%)	105 (69.1%)	0.826
Statin	269 (86.8%)	124 (81.6%)	0.141

LM: left main; LAD: left anterior descending; RI: ramus intermedius; LCx: left circumflex; RCA: right coronary artery; ADP: adenosine diphosphate; ACEI: angiotensin converting enzyme inhibitor; ARB: angiotensin receptor blocker. BMI: body mass index; CAD: coronary artery disease; SA: stable angina; UA: unstable angina; NSTEMI: non-ST elevation myocardial infarction; STEMI: ST elevation myocardial infarction; TC: total cholesterol; TG: triglyceride; HDL: high-density lipoprotein; LDL: low-density lipoprotein.

### Cardiovascular death, myocardial infarction, and stroke in anemic patients

**Figure 1:**
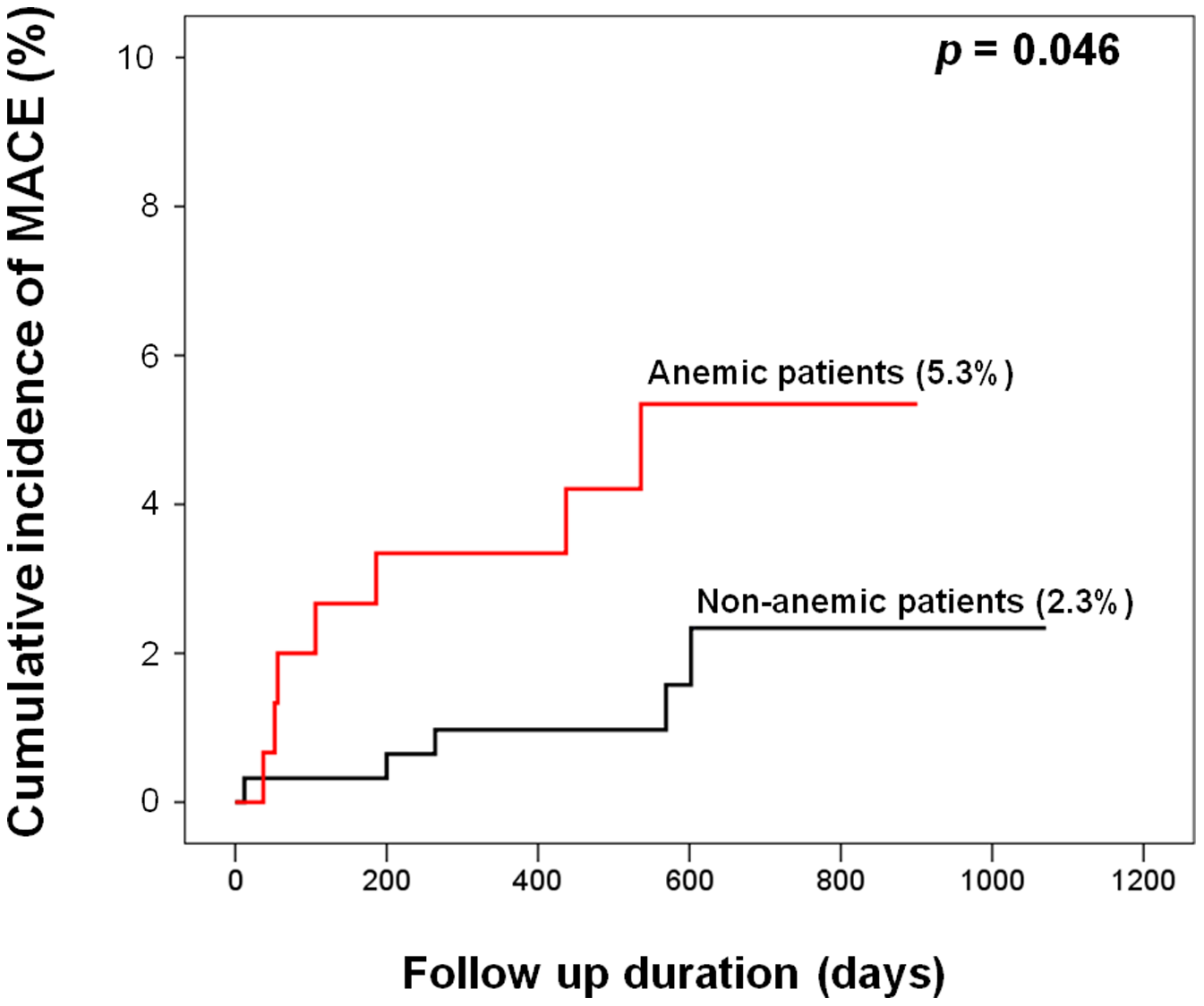
Cumulative Kaplan–Meier estimates for MACE. Anemic patients had a significantly higher event rate than non-anemic patients (*p* = 0.046). MACE: major adverse cardiac events; a composite of cardiac death, myocardial infarction, and stroke.

Before exploring the relationship between the platelet function test results and hematocrit, we first evaluated whether anemia was associated with MACE. Anemic patients demonstrated a significantly higher incidence of MACE than non-anemic patients in a Kaplan–Meier analysis (5.3% vs. 2.3%; *p* = 0.046; Figure 1).

### Association between the platelet function test results and hematocrit

**Figure 2:**
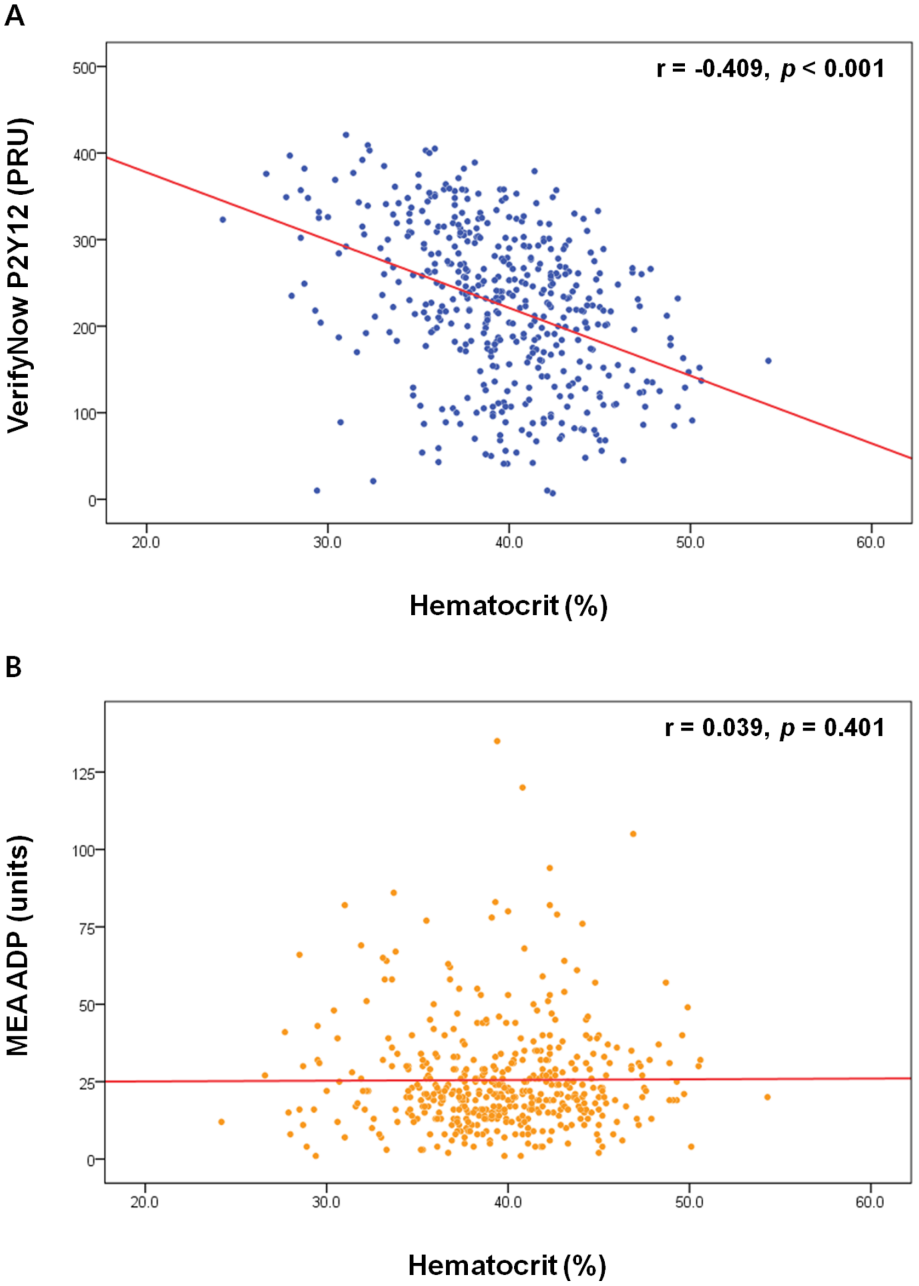
Correlation analysis between the results of platelet function tests and hematocrit. A: A negative correlation was observed between the PRU value and hematocrit. B: No significant correlation was observed between the MEA ADP assay results and hematocrit. MEA ADP: multiple electrode platelet aggregometry adenosine diphosphate test; PRU: P2Y12 reaction units.

**Figure 3:**
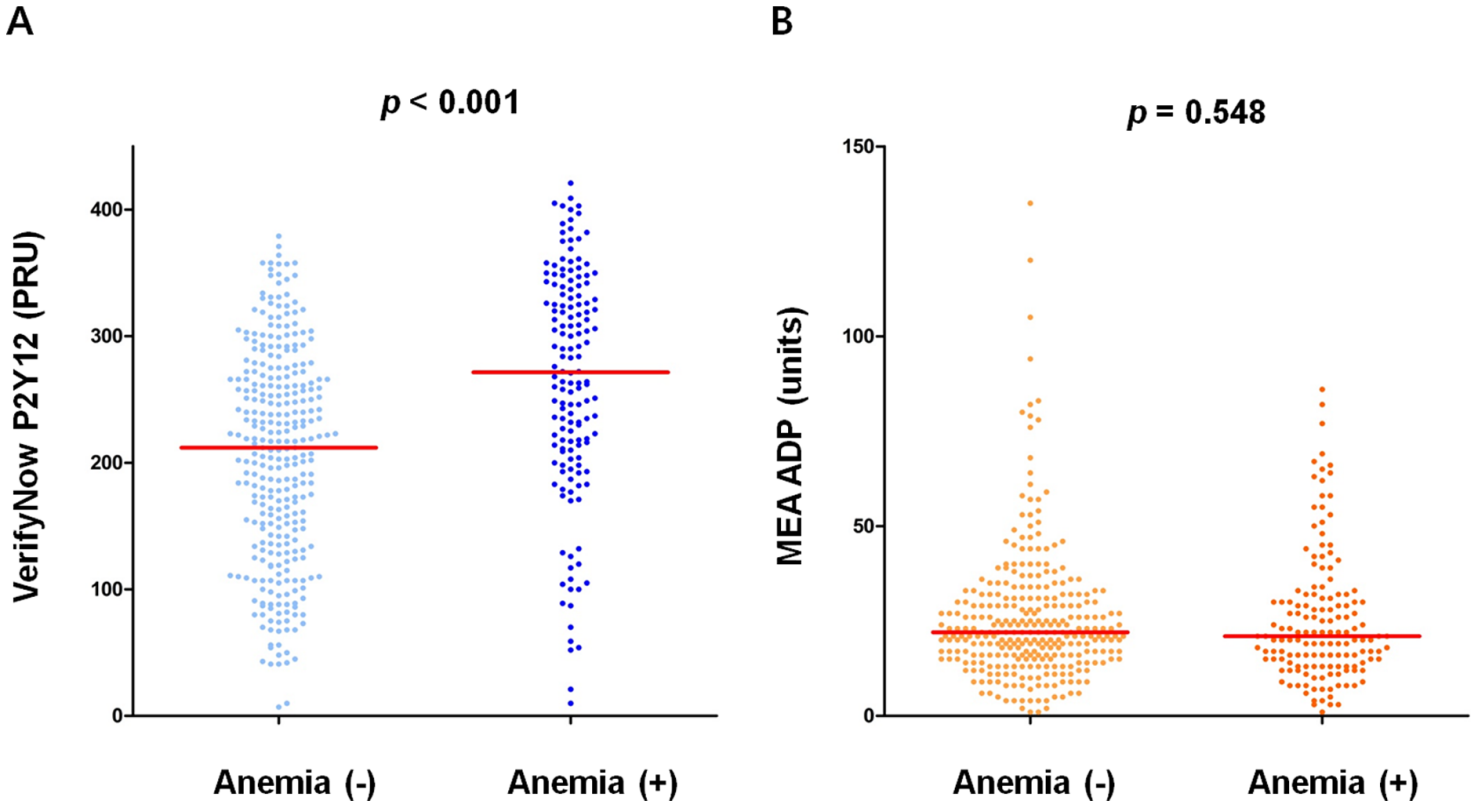
On-treatment platelet reactivity. Scatterplots of the VerifyNow P2Y12 (A) and MEA ADP (B) assays according to the presence of anemia. The red line denotes the median value. MEA ADP: multiple electrode platelet aggregometry adenosine diphosphate test.

There was a significant inverse correlation between the PRU value and hematocrit (r = −0.409; *p*<0.001; Figure 2-A). The MEA ADP assay results, however, did not correlate with hematocrit (r = 0.039; *p* = 0.401; Figure 2-B). Anemic patients had a significantly higher PRU value than non-anemic patients (271.50 [210.25–336.00] PRU vs. 212.00 [137.00–266.00] PRU; *p*<0.001; Figure 3-A; also see Figure S1 for VerifyNow % inhibition). There was no significant difference between non-anemic and anemic patients in terms of the MEA ADP assay results (21.00 [13.00–31.00] units vs. 22.00 [16.00–31.00] units; *p* = 0.548; Figure 3-B). Patients with HTPR (defined as a PRU ≥252.5) following ADP-receptor antagonist treatment had a significantly lower hematocrit level (37.68%±4.33% vs. 40.83%±4.32%; *p*<0.001; Figure S2-A). By applying PRU value of other studies performed in western population (PRU ≥240.0), [13],[14] hematocrit level was still substantially lower in patients with HTPR (37.90%±4.30% vs. 40.95%±4.37%; *p*<0.001; Figure S2-B). Hemoglobin level was also significantly lower in patients with HTPR (Figure S3).

**Figure 4:**
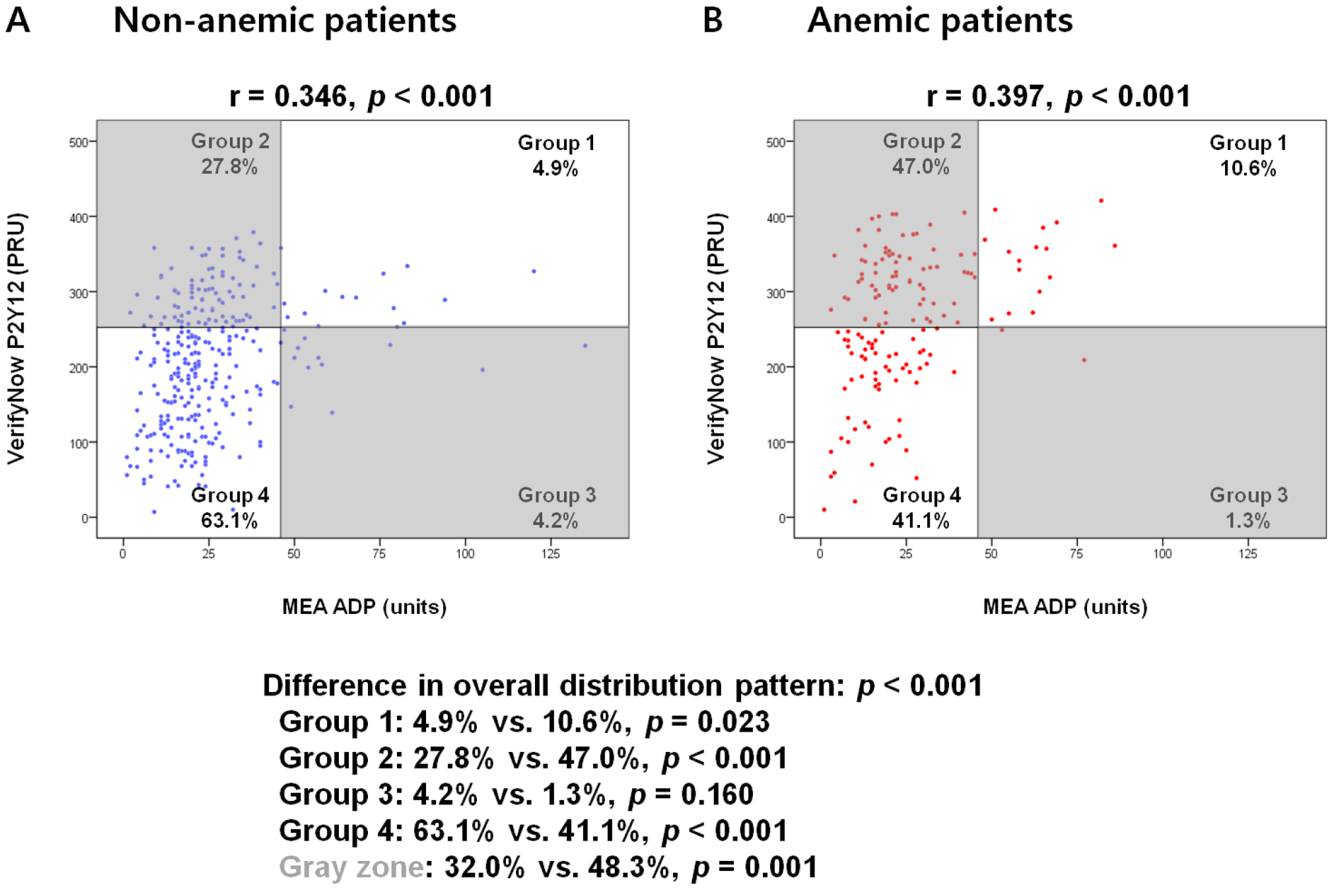
Scatter plots of the 2 point-of-care platelet function tests. The overall distribution pattern of each patient was different between the non-anemic (A) and anemic (B) patient groups. MEA ADP: multiple electrode platelet aggregometry adenosine diphosphate test; PRU: P2Y12 reaction units.

We analyzed the scatter plots of the VerifyNow P2Y12 and MEA ADP assay results separately both in non-anemic and anemic patients (Figure 4). According to the cut-off values defined in the previous study, (VerifyNow P2Y12≥252.5 [PRU], MEA ADP≥46 [units]) [1],[12] we classified each patient into 4 groups. The overall distribution pattern of each patient was considerably different between the non-anemic and anemic patient groups. Importantly, the proportion of the gray zone (where there was a discordance between the 2 point-of-care platelet function tests) was significantly different between the non-anemic and anemic patient groups (32.0% vs. 48.3%; *p* = 0.001; Figure 4). The proportion of patients with VerifyNow P2Y12≥252.5 (PRU) was 57.24% in anemic group compared to 32.36% in non-anemic group (*p*<0.001). There was no significant difference in the proportion of patients with MEA ADP≥46 (units) between the two groups (11.84% vs. 9.03%; *p* = 0.337).

### Multivariate analysis

In the multivariate analysis, anemia was independently associated with HTPR (odds ratio [OR] = 2.16, 95% confidence interval [CI] = 1.38–3.37; *p* = 0.001; Table 3) as assessed by the VerifyNow P2Y12 assay (PRU ≥252.5). The female sex (OR = 2.17, 95% CI = 1.28–3.67; *p* = 0.004) and age (OR = 1.02, 95% CI = 1.00–1.05; *p* = 0.038) were also associated with HTPR. Importantly, even after including the MEA ADP assay results in the multivariate model to account for the change in intrinsic platelet reactivity due to the ADP-receptor antagonist treatment, anemia still remained an independent predictor of HTPR (OR = 2.21, 95% CI = 1.39–3.52; *p* = 0.001). Smoking patients had a significantly higher hematocrit level (41.08%±4.30% vs. 38.21%±4.41%; *p*<0.001), and smoking status was independently associated with HTPR (OR = 0.51, 95% CI = 0.38–0.74; *p*<0.001) in univariate analysis. Smoking status, however, was not independently associated with HTPR in the multivariate model including anemic status as an independent variable.

**Table 3:** Multivariate logistic regression model used to predict HTPR after adenosine diphosphate-receptor antagonist treatment.

	Odds ratio	95% confidence interval	*p* value
Female sex	2.170	1.283–3.670	0.004
Age	1.023	1.001–1.046	0.038
Hypertension	0.991	0.626–1.569	0.969
Smoking history	0.884	0.536–1.460	0.631
BUN (mg/dL)	1.023	0.987–1.060	0.207
TC (mg/dL)	1.012	0.995–1.030	0.164
TG (mg/dL)	0.998	0.995–1.001	0.209
LDL (mg/dL)	0.985	0.966–1.004	0.123
Amlodipine	1.301	0.822–2.060	0.262
Anemia	2.157	1.382–3.368	0.001

BUN: blood urea nitrogen; HTPR: high on-treatment platelet reactivity. Other abbreviations as in Table 1. TC: total cholesterol; TG: triglyceride; LDL: low-density lipoprotein.

### Hematocrit-adjusted PRU and correlation between the 2 assays

**Figure 5:**
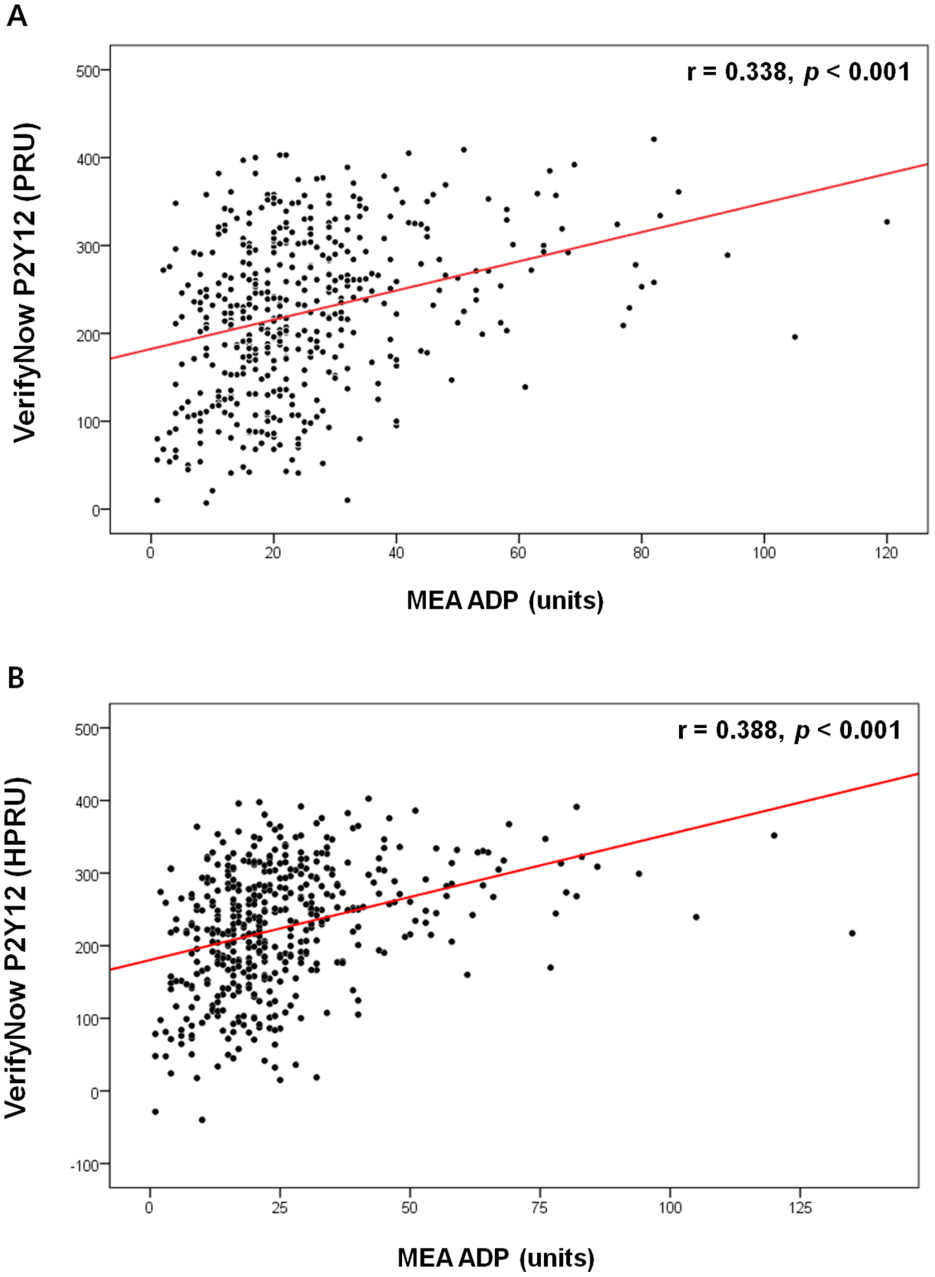
Correlation analysis between the results of the 2 point-of-care platelet function tests. A: Correlation analysis between the PRU value and MEA ADP assay results. B: Correlation analysis between the HPRU value and MEA ADP assay results. HPRU: hematocrit-adjusted PRU; MEA ADP: multiple-electrode platelet aggregometry adenosine diphosphate test; PRU: P2Y12 reaction units.

By performing both the VerifyNow P2Y12 and MEA ADP assays, we identified a moderate positive correlation between the results of the 2 tests (r = 0.338; *p*<0.001; Figure 5-A) which was in accordance with the previous report. [15] Because the VerifyNow P2Y12 assay results correlated significantly with hematocrit, we explored the utility of adjusting the PRU value for the influence of hematocrit by developing a new variable, the hematocrit-adjusted PRU (HPRU). A correction coefficient (unstandardized regression coefficient) was calculated by conducting a univariate linear regression analysis between the VerifyNow P2Y12 assay results and hematocrit. These calculated correction coefficients were −7.24 PRU for every percent increase in hematocrit levels in men and −5.56 PRU in women. The HPRU value was obtained by subtracting or adding 7.24 PRU for every percent hematocrit below or above the average hematocrit level, respectively, in men. The HPRU value for women was calculated in an identical manner. After adjustment, no correlation was found between the HPRU value and hematocrit (Figure S4). However, a significant correlation was found between the HPRU value and MEA ADP assay results (r = 0.388; *p*<0.001; Figure 5-B) and the coefficient of correlation was slightly increased (0.338 to 0.388) by adjusting the PRU value for hematocrit.

## Discussion

This study demonstrated that the VerifyNow P2Y12 assay result is significantly influenced by hematocrit, whereas the MEA ADP assay result is unaffected. A significant inverse correlation was observed between the PRU value and hematocrit, and anemic patients exhibited substantially higher PRU values than did non-anemic patients. Anemia was independently associated with HTPR (PRU≥252.5) in the multivariate analysis model. Previous study has reported the relationship between hematocrit and VerifyNow & MEA assays stimulated with arachidonic acid. [16] We report herein the relationship between hematocrit and VerifyNow & MEA assays stimulated with ADP. To our knowledge, this is the first clinical study to simultaneously evaluate the effect of hematocrit on the results of both the VerifyNow P2Y12 and MEA ADP assays.

### Different platelet function tests and their limitations

The VerifyNow and MEA assays differ from light transmission aggregometry (LTA), the gold standard, in that they use whole blood rather than platelet-rich plasma, thus eliminating the need for centrifugation. This advantage over LTA makes them to function as point-of-care assays. [1] The MEA assay differs from the VerifyNow assay in that platelet aggregation occurs on a surface rather than in a liquid phase which is a process similar to in-vivo platelet aggregation, during which platelet adhesion and aggregation occur on a ruptured plaque or injured vessel wall. No platelet function test, however, has been found superior to the others in terms of predicting atherothrombotic complications in large-scale clinical trials. Based on our study finding that the VerifyNow P2Y12 and MEA ADP assay results only moderately correlated with each other (r = 0.338, *p*<0.001), we can assume that both tests require further improvements in order to better evaluate the actual in-vivo platelet functions of patients.

### Association between the VerifyNow P2Y12 assay results and hematocrit: the mechanism

The observed inverse correlation between the PRU value and hematocrit might represent either a true in-vivo effect of hematocrit on platelet reactivity or a mere laboratory artifact. Previous report has suggested that HTPR in anemic patients is one of the mechanisms for increased atherothrombotic events in anemic patients. [5] However, Kakouros et al. reported that the association between the PRU value and hematocrit was more likely an in-vitro phenomenon inherent to the assay. [2] Interestingly, in that article, hematocrit was independently associated with the PRU value in a multivariate analysis model, even after including LTA in the model to account for the ADP-receptor antagonist-induced change in intrinsic platelet reactivity. A similar result was noted in our study when the MEA ADP assay was included instead of LTA. If this association is indeed an in-vitro laboratory artifact, the hematocrit level should be seriously considered when interpreting VerifyNow P2Y12 assay results.

The VerifyNow P2Y12 system measures platelet reactivity by calculating changes in the blood sample turbidity after stimulating platelet aggregation with ADP; specifically, the light transmission increases as the activated platelets aggregate. This instrument measures the change in the optical signal and reports the results in PRU. Although the exact algorithm used to calculate this PRU value is not known, we assume that the change in turbidity might be exaggerated in blood samples with few red blood cells because baseline turbidity is low in such samples, and, therefore, a small change in turbidity due to ADP stimulation might result in a great percentile change in the overall turbidity. On the other hand, in blood samples with abundant red blood cells, any ADP stimulation-related change in turbidity would not translate into a significant overall change in turbidity because the baseline turbidity is already high. The exact mechanism, however, has not been fully determined and requires further investigation.

### Association between the VerifyNow P2Y12 assay results and hematocrit: the clinical implication

A substantial proportion of patients who present with CAD are anemic (32.9% in this study). Furthermore, substantial proportion of anemic patients had HTPR according to PRU value but showed normal MEA ADP assay results in this study (47.0%; Figure 4). An unadjusted PRU value could possibly lead to treating anemic patients, who has high PRU values but normal in-vivo platelet reactivity, with unnecessary intensified antiplatelet regimens. The opposite scenario is also possible.

To date, no clinical trials have demonstrated an improved clinical outcome associated with individualized antiplatelet therapy according to platelet function tests. The ARCTIC, [17] GRAVITAS, [18] and TRIGGER-PCI [19] randomized trials were intended to evaluate the effects of intensified antiplatelet therapy in patients with HTPR as measured using the VerifyNow P2Y12 assay. All 3 trials have failed to prove an advantage of these intensified antiplatelet regimens. However, because the VerifyNow P2Y12 assay is hematocrit dependent and a substantial proportion of the patients presenting with CAD are anemic, the results of these studies might have been confounded by the influence of hematocrit. Adjusting for the effect of hematocrit or performing a simultaneous MEA ADP assay might better classify the patient group that would most benefit from an intensified antiplatelet regimen, a possibility that should be investigated in future clinical trials.

### Limitations

The gold standard LTA was not performed in this study. However, we intended to compare the point-of-care platelet function tests that could be applied in actual clinical practice to provide rapid evaluations of residual platelet function. Relatively small number of patients is another limitation of this study and this study was not powered to assess the clinical outcome in relation to the presence of anemia. Finally, pharmacokinetic data to evaluate the correlation between platelet function test results and plasma concentration of clopidogrel active metabolite are not available.

### Conclusions

Hematocrit level significantly influences the VerifyNow P2Y12 assay results, representing a presumed in-vitro artifact. Correlation between the VerifyNow P2Y12 and MEA ADP assays was improved by adjusting for the influence of hematocrit. Adjusting PRU for hematocrit or simultaneously performing the MEA ADP assay might help to better guide antiplatelet therapy.

## Supporting Information

Figure S1 **Difference in the VerifyNow P2Y12 % inhibition according to the presence of anemia.** Patients with anemia showed a significantly lower VerifyNow P2Y12 % inhibition level compared to the non-anemic patients (43.00 [26.00–63.00] % vs. 19.00 [9.75–34.75] %; *p*<0.001).
(TIF)

Figure S2 **Differences in the hematocrit level according to the presence of HTPR.** The patient group with HTPR after ADP-receptor antagonist treatment (A: PRU≥252.5, B: PRU≥240.0) exhibited a significantly lower hematocrit level. ADP: adenosine diphosphate; HTPR: high on-treatment platelet reactivity; PRU: P2Y12 reaction units.
(TIF)

Figure S3 **Difference in the hemoglobin level according to the presence of HTPR.** Hemoglobin level was significantly lower in patients with HTPR (PRU≥252.5). HTPR: high on-treatment platelet reactivity; PRU: P2Y12 reaction units.
(TIF)

Figure S4 **Correlation analysis between the HPRU value and hematocrit.** After adjusting for the influence of hematocrit, the association between the VerifyNow P2Y12 assay results and hematocrit was disappeared both in men (A) and women (B). HPRU: hematocrit-adjusted P2Y12 reaction units.
(TIF)
