# Supplementary figures and images for: Different Influences of Hematocrit on the Results of Two Point-Of-Care Platelet Function Tests, the VerifyNow Assay and Multiple Electrode Platelet Aggregometry
Source: PLoS One. 2014 Nov 26;9(11):e114053. doi: 10.1371/journal.pone.0114053 (PMC4245259; doi:10.1371/journal.pone.0114053)

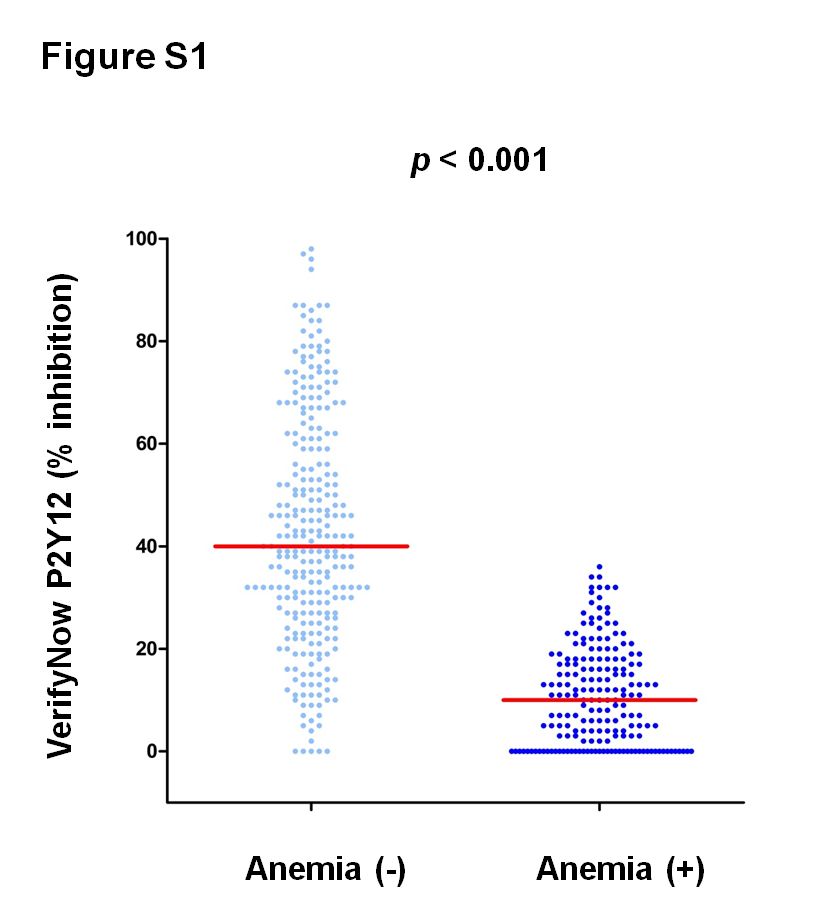

Supplement: Figure S1 — Difference in the VerifyNow P2Y12 % inhibition according to the presence of anemia. Patients with anemia showed a significantly lower VerifyNow P2Y12 % inhibition level compared to the non-anemic patients (43.00 [26.00–63.00] % vs. 19.00 [9.75–34.75] %; p<0.001). (TIF) [file pone.0114053.s001.tif]

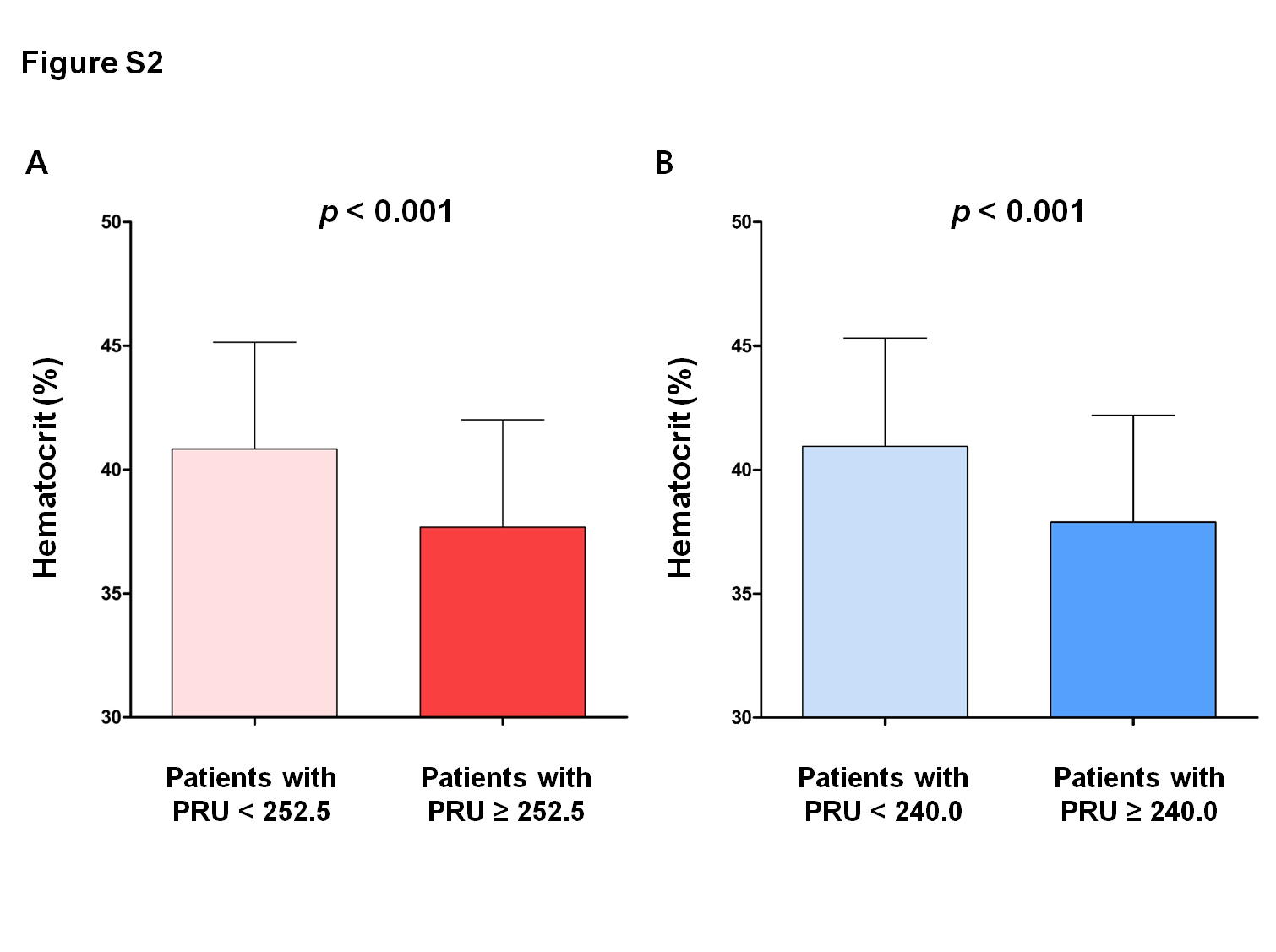

Supplement: Figure S2 — Differences in the hematocrit level according to the presence of HTPR. The patient group with HTPR after ADP-receptor antagonist treatment (A: PRU≥252.5, B: PRU≥240.0) exhibited a significantly lower hematocrit level. ADP: adenosine diphosphate; HTPR: high on-treatment platelet reactivity; PRU: P2Y12 reaction units. (TIF) [file pone.0114053.s002.tif]

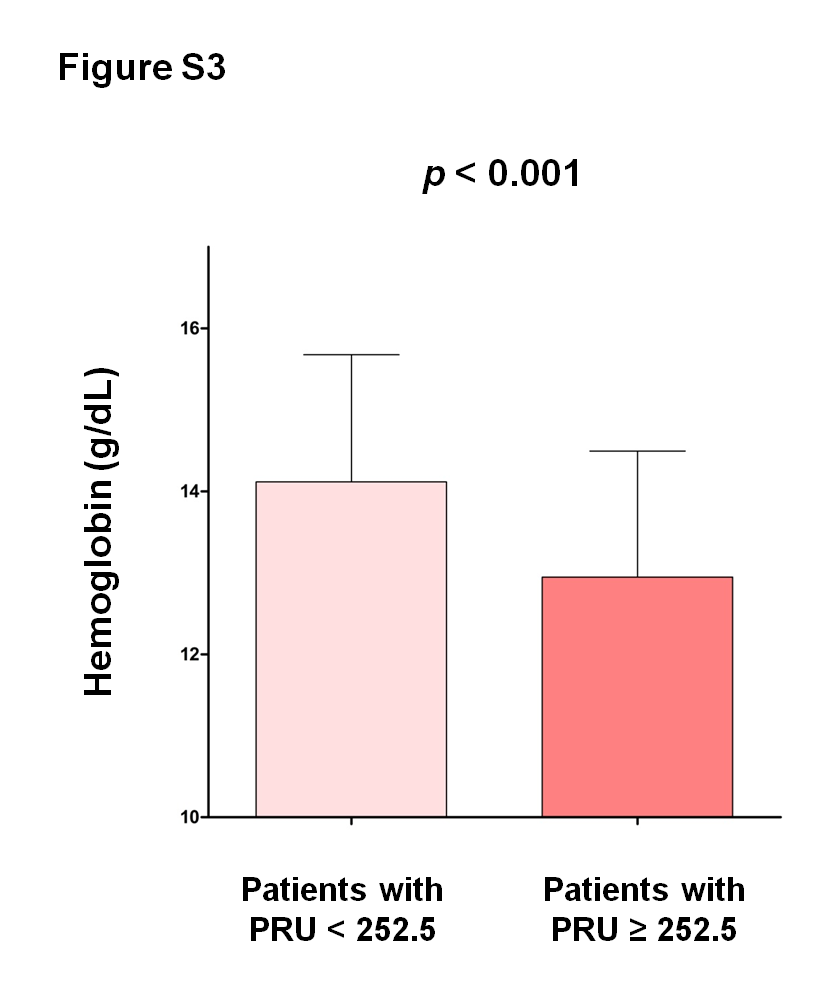

Supplement: Figure S3 — Difference in the hemoglobin level according to the presence of HTPR. Hemoglobin level was significantly lower in patients with HTPR (PRU≥252.5). HTPR: high on-treatment platelet reactivity; PRU: P2Y12 reaction units. (TIF) [file pone.0114053.s003.tif]

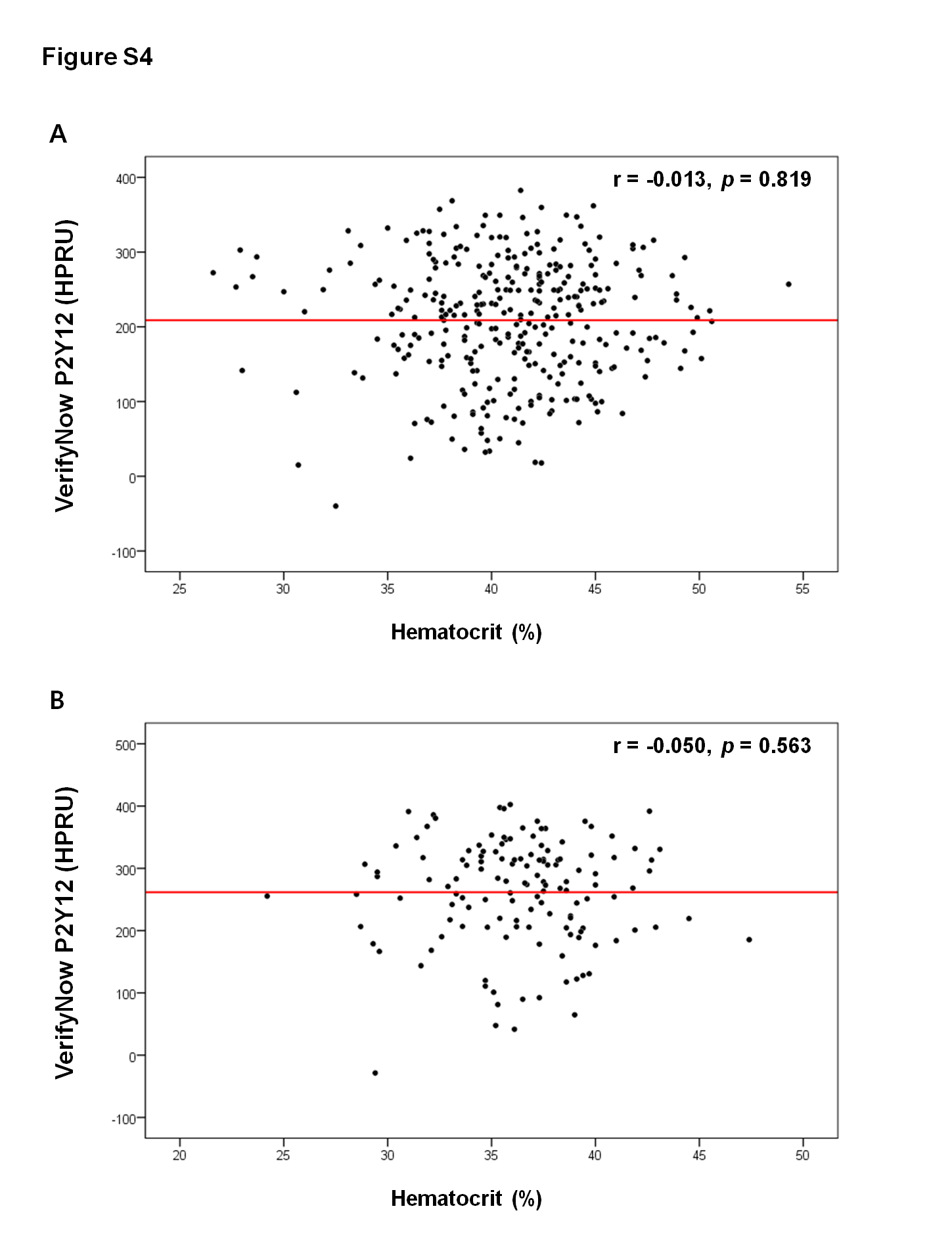

Supplement: Figure S4 — Correlation analysis between the HPRU value and hematocrit. After adjusting for the influence of hematocrit, the association between the VerifyNow P2Y12 assay results and hematocrit was disappeared both in men (A) and women (B). HPRU: hematocrit-adjusted P2Y12 reaction units. (TIF) [file pone.0114053.s004.tif]
